# A Unique Pathogenic Heterozygous Hypoxia Up-Regulated Protein 1 (HYOU1) Mutation Presenting With Recurrent Osteomyelitis, Subglottic Stenosis, and Hypoglycemia

**DOI:** 10.7759/cureus.100028

**Published:** 2025-12-24

**Authors:** Lauren Worth, Remie Saab, Sarah L Azzi, Gabrielle Tan, Robert Hostoffer

**Affiliations:** 1 Allergy and Immunology, University Hospitals Cleveland Medical Center, Cleveland, USA; 2 Allergy and Immunology, Lake Erie College of Osteopathic Medicine, Erie, USA; 3 Allergy and Immunology, Case Western Reserve University, Cleveland, USA

**Keywords:** hyou1, hypoglycemia, hypoxia up-regulated protein 1, mutation, recurrent infections, subglottic stenosis

## Abstract

The hypoxia up-regulated protein 1 (HYOU1) gene encodes a chaperone protein involved in maintaining cellular homeostasis by reducing oxidative stress and supporting the unfolded protein response in the endoplasmic reticulum and mitochondria. While homozygous HYOU1 mutations have been previously reported in patients with profound immunodeficiency, pancytopenia, and hypoglycemia, the clinical implications of heterozygous mutations remain less defined. We describe a patient with a heterozygous pathogenic HYOU1 mutation who presents with a longstanding history of recurrent infections, medically treated hypoglycemia, and subglottic stenosis due to repeated intubation and inflammation.

## Introduction

Several genetic mutations have been linked to recurrent infections that vary in severity from mild to life-threatening. The hypoxia up-regulated protein 1 (HYOU1) is a gene that encodes a chaperone protein that is involved in mediating cell stress within the endoplasmic reticulum and mitochondria [1]. The mechanism of this protein is accomplished by reducing oxidative stress in cells and aiding in unfolded protein responses. Mutations in the HYOU1 gene cause converging defects, including impaired B-cell maturation, reduced T-cell activation under stress, increased susceptibility of immune cell death during infection, and defective antigen presentation. This mutation, although not lethal initially, is associated with infections in various organ systems, causing end-organ damage with subsequent lethality [2,3]. Careful evaluation made by astute clinical questioning will lead to a diagnosis and treatment of infections, as well as the implementation of prophylactic strategies. 

A previous case report identified a homozygous HYOU1 mutation that presented with anemia, thrombocytopenia, and leukopenia, resulting in severe immunodeficiency, repeated infections, and ultimately death [4]. Another case of an HYOU1 mutation reports a 45-year-old woman with recurrent infections and bouts of hypoglycemia throughout her life [2]. Our patient presented for shortness of breath, which was determined to be due to subglottic stenosis. We were able to ascertain a history of chronic infections and osteomyelitis, hypoglycemia, and later a heterozygous genetic mutation in HYOU1 (exon 23, c.2638G>A) (p.Ala880Thr). We present the first case of a patient with an HYOU1 mutation associated with recurrent osteomyelitis and subglottic stenosis.

A preliminary version of this work was previously presented as an abstract at the Journal of Human Immunology in Philadelphia, Pennsylvania, in 2025.

## Case presentation

**Figure 1:**
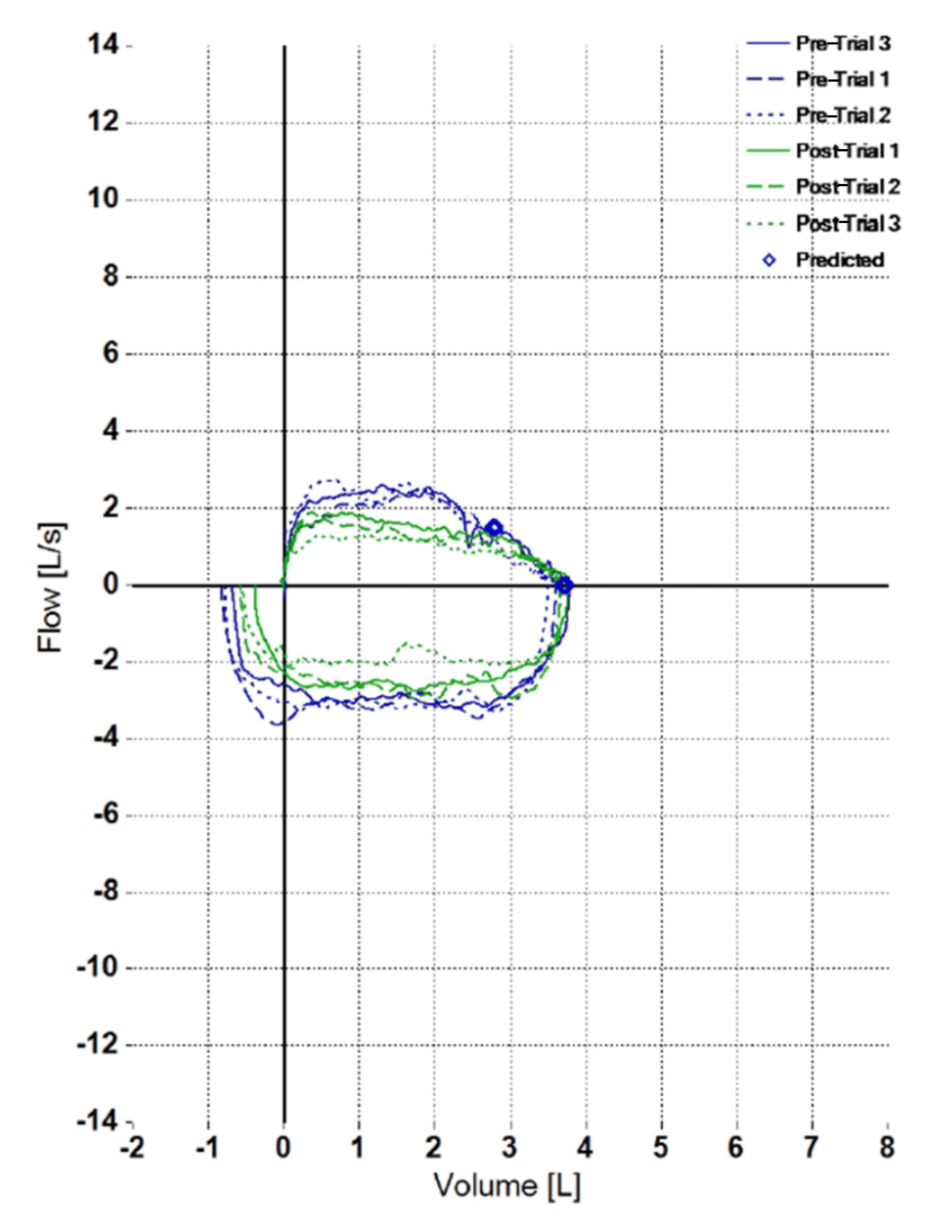
Pulmonary function test displaying fixed airway obstruction.

A 32-year-old female patient presented with chronic shortness of breath, a history of recurrent osteomyelitis, chronic sinusitis, and medically managed hypoglycemia. She experienced frequent sinus infections that responded to antibiotics. Her medical history also included polycystic ovarian syndrome and attention-deficit disorder. She was using a triple inhaler (inhaled corticosteroid/long-acting beta-agonist/long-acting muscarinic antagonist (ICS/LABA/LAMA)) for patient-described dyspnea, which she reported improved her symptoms. The patient's history included 20 surgeries with frequent intubations due to osteomyelitis involving the lower extremities, including the femur. Recurrent osteomyelitis has been observed in individuals with underlying immunodeficiencies [5]. Her family history is notable for hypoglycemia, arthritis, and Hashimoto's thyroiditis. A pulmonary function test, which can be used to evaluate upper airway obstructions such as subglottic stenosis, was performed to investigate her shortness of breath [6]. The test demonstrated intrathoracic obstruction (Figure 1). 

**Figure 2:**
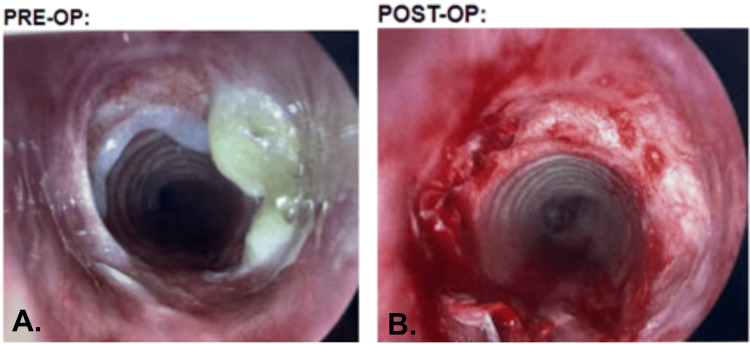
(A) Subglottic stenosis of 15%, located at 1.8 cm below the vocal folds, with yellow secretions. (B) Post-dilation with a CRE balloon and Kenalog injection. CRE: controlled radial expansion

She underwent an immunodeficiency evaluation to determine a potential cause of her recurrent infections and a genetic evaluation. She was referred to the ear, nose, and throat (ENT), who diagnosed her with subglottic stenosis, due to chronic infections and intubations, which was deemed untreatable surgically (Figure 2). Subglottic stenosis can be caused by repetitive intubation and chronic infection, which were present in this patient's history [7]. Cultures were taken from the infection site, and appropriate antibiotics were initiated.

Investigative results indicated a heterozygous pathogenic mutation in the HYOU1 gene (exon 23, c.2638G>A) (p.Ala880Thr), normal immunoglobulin levels, lymphocyte subpopulations, and neutrophil oxidative burst assay within normal ranges. A complete blood count (CBC) and basic metabolic panel (BMP) were unremarkable. Cultures from a laryngeal biopsy were positive for several microorganisms, including *Klebsiella* and methicillin-resistant *Staphylococcus aureus* (MRSA), which was sensitive to ciprofloxacin (Table 1) [8,9]. Despite effective treatment courses with ciprofloxacin, the infection continued to return. The patient was started on prophylactic ciprofloxacin due to failure of other prophylactic therapies. Since the initiation of prophylaxis, she has remained clinically stable, with no recurrence of osteomyelitis or infections involving the subglottic region.

**Table 1:** Culture report from the laryngeal biopsy.

Swab-larynx biopsy specimen	
Component	6 months ago
Culture, wound	Many *Klebsiella (Enterobacter) aerogenes*
Culture, wound	Few methicillin-resistant *Staphylococcus aureus*
Culture, wound	Few normal respiratory flora
Gram stain	Many Gram-positive cocci
Gram stain	Many Gram-negative bacilli
Gram stain	Few Gram-positive bacilli
Gram stain	Rare polymorphonuclear leukocytes

## Discussion

Patients with homozygous mutations in HYOU1 have previously presented with hypoglycemia and severe, life-threatening immunodeficiencies. The HYOU1 gene encodes a chaperone protein involved in cell homeostasis, located in the endoplasmic reticulum and mitochondria [1]. Our patient presented with shortness of breath, and it was later elicited from history that she had chronic infections and hypoglycemia. This constellation of symptoms prompted pulmonary function testing and an immunodeficiency evaluation to investigate the cause of her recurrent infections. Although the immunology panel returned normal results, genetic testing revealed a heterozygous mutation in HYOU1. This genetic finding led to a review of previous cases with homozygous HYOU1 mutations, which showed a similar collection of symptoms. Previous reports describe patients with HYOU1 mutations presenting with combined immunodeficiency and disrupted glucose metabolism, aligning with the hypoglycemia observed in our patient [2]. Her pulmonary function test indicated fixed intrathoracic obstruction, which was supported by laryngoscopy revealing a subglottic stenosis (Figure 2).

## Conclusions

We report a novel heterozygous HYOU1 mutation (exon 23, c.2638G>A) (p.Ala880Thr) associated with several pathologies in a 32-year-old woman. Given the patient's history of the genetic mutation, we propose that her recurrent osteomyelitis, infectious subglottic stenosis, and medically treated hypoglycemia are correlated to her pathogenic heterozygous HYOU1 mutation (exon 23, c.2638G>A) (p.Ala880Thr). The HYOU1 gene encodes a chaperone protein involved in cell homeostasis, located in the endoplasmic reticulum and mitochondria. Mutations involved with this gene have been linked to several pathologies in previous literature, such as hypoglycemia and severe, life-threatening immunodeficiencies. Healthcare providers should be aware of the possibility of this novel HYOU1 mutation (exon 23, c.2638G>A) (p.Ala880Thr) being associated with various pathologies, such as those in our patient. Increased awareness of this potential association may aid clinicians in recognizing atypical presentations and guiding future investigation.
